# Microbial Assemblages in Pressurized Antarctic Brine Pockets (Tarn Flat, Northern Victoria Land): A Hotspot of Biodiversity and Activity

**DOI:** 10.3390/microorganisms7090333

**Published:** 2019-09-09

**Authors:** Maria Papale, Angelina Lo Giudice, Antonella Conte, Carmen Rizzo, Alessandro C. Rappazzo, Giovanna Maimone, Gabriella Caruso, Rosabruna La Ferla, Maurizio Azzaro, Concetta Gugliandolo, Rodolfo Paranhos, Anderson S. Cabral, Vincenzo Romano Spica, Mauro Guglielmin

**Affiliations:** 1 Institute of Polar Sciences, National Research Council (ISP-CNR), 98122 Messina, Italy (M.P.) (A.C.R.) (G.M.) (G.C.) (R.L.F.) (M.A.); 2 Department of Chemical, Biological, Pharmaceutical and Environmental Sciences, University of Messina, 98168 Messina, Italy (A.C.) (C.R.) (C.G.); 3 Institute of Biology, Federal University of Rio de Janeiro, Rio de Janeiro 21.941-590, Brazil (R.P.) (A.S.C.); 4 Department of Movement, Human and Health Sciences, Public Health Unit, University of Rome “Foro Italico”, P.zza Lauro De Bosis 6, 00135 Rome, Italy; 5 Dipartimento di Scienze Teoriche e Applicate, University of Insubria, 21100 Varese, Italy

**Keywords:** brine pockets, hyperthermophiles, sulphur-reducing bacteria, methanogens, metabolic potential

## Abstract

Two distinct pressurized hypersaline brine pockets (named TF4 and TF5), separated by a thin ice layer, were detected below an ice-sealed Antarctic lake. Prokaryotic (bacterial and archaeal) diversity, abundances (including virus-like particles) and metabolic profiles were investigated by an integrated approach, including traditional and new-generation methods. Although similar diversity indices were computed for both Bacteria and Archaea, distinct bacterial and archaeal assemblages were observed. Bacteroidetes and Gammaproteobacteria were more abundant in the shallowest brine pocket, TF4, and Deltaproteobacteria, mainly represented by versatile sulphate-reducing bacteria, dominated in the deepest, TF5. The detection of sulphate-reducing bacteria and methanogenic Archaea likely reflects the presence of a distinct synthrophic consortium in TF5. Surprisingly, members assigned to hyperthermophilic Crenarchaeota and Euryarchaeota were common to both brines, indicating that these cold habitats host the most thermally tolerant Archaea. The patterns of microbial communities were different, coherently with the observed microbiological diversity between TF4 and TF5 brines. Both the influence exerted by upward movement of saline brines from a sub-surface anoxic system and the possible occurrence of an ancient ice remnant from the Ross Ice Shelf were the likely main factors shaping the microbial communities.

## 1. Introduction

In continental Antarctica, brines are hypersaline solutions generally found within permafrost [1,2,3]. In recent years, they have been discovered also within glaciers [4], and below ice-sealed and subglacial lakes [5,6]. Both the genesis and mobilization of brines within permafrost and glaciers, as well as below lakes, are largely unknown [7]. The high salt content of Antarctic brines is responsible for the maintenance of their unfrozen condition at several degrees below 0 °C. 

One would imagine that life under these incredibly harsh environmental conditions is sporadic and in a suspended state, but this is not the case of Antarctic brines, where microorganisms can become dominant in terms of biodiversity, biomass and activity [8]. Cold-adapted microorganisms, classically distinguished into psychrophiles and psychrotrophs, depending on their optimal temperature and temperature range for growth [9], surmount the negative effects of low temperatures (also occurring together with additional environmental constraints) by adopting survival strategies based on a number of structural and physiological modifications (see [10], and references therein). Some well-known examples are the modulation of the fatty acid chain length, the proportion of *cis* to *trans* fatty acids and the type of carotenoids in cell membranes (for the maintenance of membrane fluidity), an appropriate rate for enzyme-catalyzed reactions (e.g., primary cell processes such as transcription and translation), the synthesis of cold-shock, cold-acclimation and heat-shock proteins, and the production of cryoprotective compounds (e.g., extracellular polymeric substances and polyhydroxyalkanoates, antifreeze and antinucleating proteins) [11,12,13,14]. All of those peculiar features make cold-adapted microorganisms particularly interesting as underexplored sources of novel bioactive molecules for biotechnological purposes [10]. Furthermore, the discovery of brines in the sub-surfaces of Mars and Jupiter’s moon Europa makes Antarctic brines attractive as terrestrial analogue counterparts and potential astrobiological targets [15]. The occurrence of such liquids is central to understand the potential for extant life on the Red Planet [16], and the exploration of psychrophiles on Earth thus becomes particularly significant for our comprehension of the boundaries of life on Earth or elsewhere in our solar system [5,17,18,19,20,21]. This is also true if considering methanogens that are thought to have been the first autotrophs to evolve on Earth, playing a crucial role in the early evolution of our biosphere. Such chemoautotrophs utilize molecular hydrogen and carbon dioxide and produce methane as a waste product, thus making conceivable, their existence on Mars [22].

Recently, Forte et al. [23] detected, through ground probing radar (GPR) surveys, two distinct pressurized hypersaline brine pockets, separated by a thin ice layer, below an ice-sealed Antarctic lake in the highest elevation area of Tarn Flat (Northern Victoria Land). These brines were located within a pingo-like-feature (PLF) [23], similar to those identified on Mars [15]. Forte et al. [23] demonstrated that the mound was formed by the extrusion of brines, possibly from underground taliks (external to the lake basin), aligned along fractures (or faults) oriented NNW–SSE. In these brines, the presence of phylogenetically different fungal communities, including various specific taxonomical groups, was reported [24]. This observed phylogenetic divergence was suggested to be strictly related to the different chemical and physical features of the two brines (especially in terms of salinity, total organic carbon, TOC, and total inorganic carbon, TIC), probably due to the physical barrier produced by the presence of the thin ice layer.

The present study was aimed at extending the current knowledge on the microbial life in the Tarn Flat brines by investigating prokaryotic (bacterial and archaeal) diversity, abundances (including virus-like particles) and metabolic potentials (e.g., enzymatic activities and physiological profiles). An integrated approach, including traditional and new-generation methods was adopted.

## 2. Materials and Methods 

### 2.1. Area and Site Description

The Tarn Flat (TF) area is the largest ice-free area of the Northern Victoria Land, located near the Italian Antarctic Research Station “Mario Zucchelli” (MZS). The presence of Late Wisconsin’s glacial deposits and outcropping Ordovician granites in the area was previous described [25,26]. The mean annual air temperature (MAAT)—based on observations over the last 20 years—is approximately −14°C, while snow ranges between 100 and 200 mm per year [27,28]. Many lakes characterize the study area and, generally, those located at higher elevations(more than 250 m a.s.l.) are frozen throughout the year or only partially melted during warmer seasons [29].

### 2.2. Sample Collection

**Figure 1:**
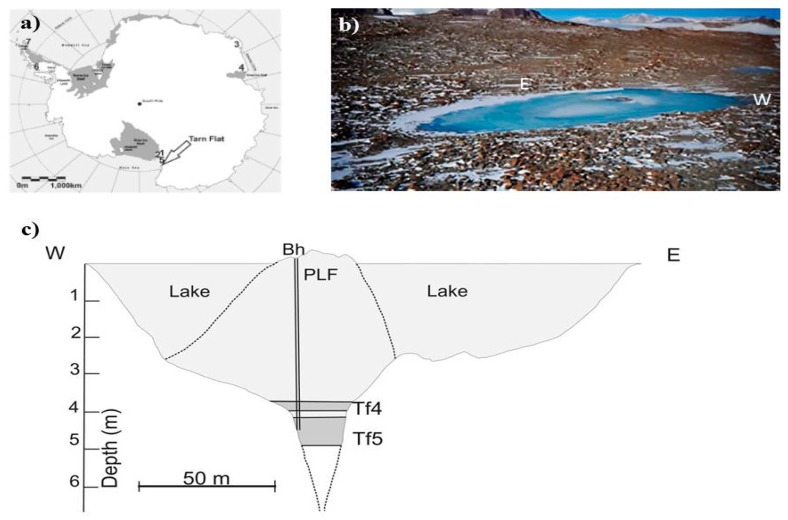
Study area: (**a**) Tarn Flat location; (**b**) a view of the Tarn Flat, its pingo-like feature (PLF) in the middle (photo courtesy by Michele Dalle Fratte and modified), and the extremes of the section reported in the next panel; (**c**) Lake section. Legend: light grey: lake and PLF ice; grey: hypersaline brines; dashed lines refer to the slope of PLF and the edges of the fracture located underneath the PLF deducted by GPR analyses by Forte et al. [23].

Brines were newly detected in 2014 in a perennially frozen lake (Lake-1 in Forte et al. [23]; Figure 1), elongated in the east–west direction and located in the highest elevation area of Tarn Flat. Sub-surface brines occurred in the deepest and central part of the lake, along a NNW–SSE axis, which corresponded to a PLF on the lake surface. This PLF reached a maximum height of 45 cm and extended within an area of approximately 500 m^2^ [23]. Due to sampling logistic issues only two samples, namely TF4 (between 3.78 and 3.98 m depth) and TF5 (between 4.10 and 4.94 m depth), were collected by a sterilized peristaltic pump and tubing. After collection, the samples were kept at −20°C at MZS until processing in Italy. The complete chemical characterization of the two brine samples has been reported by Borruso et al. [24]. Briefly, the brines were chemically different despite their proximity (only 12 cm of an ice layer divided the two pockets). TF4 showed a higher salinity than TF5 (90 and 75 psu, respectively) [23], but above all, TOC (102.5 versus 82.0 mg L^−1^) and TIC (674.8 versus 628.3 mg L^−1^) were higher in TF5 than TF4. In addition, the TF5 brine was very rich in gas bubbles strongly smelling of hydrogen sulphide, but unfortunately it was not possible to collect and analyze them.

### 2.3. Microbial Cell Abundance

#### 2.3.1. Virus-Like Particles and Prokaryotic Cells by Flow Cytometry

Samples for determining virus-like particle (VLP) abundance by flow cytometry (FC) were fixed with 0.05% glutaraldehyde, and frozen in liquid nitrogen until analysis [30]. Samples for the evaluation of the prokaryotic cell (PA^C^) abundance by FC were fixed with paraformaldehyde, 1% + glutaraldehyde, 0.05%, and frozen in liquid nitrogen until analysis [31]. VLP and PA^C^ sample aliquots were stained with SYBR Green I (5 × 10^–5^of the commercial stock solution; Molecular Probes) and analyzed using a FACSCalibur flow cytometer (BD Biosciences) equipped with a 488 nm Argon laser. To discriminate VLP and PA^C^, bivariate plots of side scatter light (SSC; related to size and morphology) and relative fluorescence (green fluorescence) were used. Based on differences in these plots, it was possible to discriminate VLP from bacterial cells, even the smallest [32]. Besides that, relative fluorescence intensity allows the discrimination of different viral clusters and highly and less active prokaryotic cells by their high (HNA) and low nucleic acid (LNA) contents, respectively [31,33,34].

#### 2.3.2. Prokaryotic Cell Abundance (PA), Morphometry, Morphology and Biomass by Image Analysis

Samples were collected in sterile 50 mL-polyethylene tubes, immediately fixed with filter-sterilized formaldehyde (2%, final concentration) and stored at 4 °C in the dark. Three replicates per brine were filtered through polycarbonate black membranes (porosity 0.22 µm; GE Water and Process Technologies) and stained for 10–20 min with DAPI (final concentration 10 µg mL^−1^) according to Porter and Feig [35]. The prokaryotic cells were quantified as described in La Ferla et al. [36,37]. The volume (VOL)of individual cells, the mean cell VOL and the total biovolume were determined as reported by La Ferla and co-authors [37]. The cell VOL was converted into cell carbon content (CCC, expressed in fg C cell^−1^) by the allometric relation proposed by Loferer-Krößbacher et al. [38]. The prokaryotic biomass (PB, µg C L^−1^) was calculated by multiplying PA to CCC derived in each sample, as described by La Ferla et al. [36]. The cell shapes were operationally defined as (i) cocci (length and width differed by less than 0.10 μm), (ii) coccobacilli (length and width differed by more than 0.10 μm), and (iii) rods (length was at least double that of their width). Vibrios, curved rods and spirillae were defined as V-shaped, C-shaped and S-shaped cells, respectively.

### 2.4. Prokaryotic Cell Viability and Respiring Rates

#### 2.4.1. Live/Dead Cells

The viability of prokaryotic cells—in terms of membrane integrity—was analyzed using the Live/Dead Bac Light Bacterial Viability Kit (Molecular Probes™) as described by La Ferla et al. [39]. The cells were counted under a Zeiss Axioplan epifluorescence microscope equipped with the filter sets specific for fluorescein (BP450–490; FT510; LP520) and rhodamine (BP546/12; FT580; LP590). All the measurements were done under aerobic conditions.

#### 2.4.2. Respiring Cells (CTC+)

To quantify the prokaryotic respiring cells, the Bac Light Redox Sensor CTC Vitality Kit (Molecular Probes^TM^) was used. The procedure was performed according to La Ferla et al. [39]. All the measurements were done under aerobic conditions.

### 2.5. Microbial Metabolic Potential and Enzymatic Activities

#### 2.5.1. Physiological Profiles

Differences in metabolic potential of microbial assemblages were determined by using Biolog-EcoPlate™ microplates, according to standard procedures [40]. 96-well microtiter plates containing 31 carbon sources and a control in triplicate together with the redox dye tetrazolium violet, were inoculated with 150 μL of each natural sample. Each plate was incubated at 4 °C in the dark under aerobic conditions [39]. The absorbance of formazan produced by oxidation processes was quantified at 590 nm using a microplate-reader spectrophotometer (MICROTITER ELX-808, Bio Whittaker, Inc.) equipped with an automatic microplate reader and the specific software (WIN KQCL) for data processing. Optical density (OD) was immediately measured after inoculation (at time 0) and once a day for a month.

The color development for each plate was expressed as averaged substrate color development (ASCD); i.e., ASCD = Σ ((R-C)/31), where R is the absorbance value averaged from three wells with substrate and C is absorbance value averaged from the control wells (without substrate) [41]. The substrate richness (catabolic richness, S) values, (i.e., total number of oxidized C substrates = total number of wells with absorbance over 0.10 OD) and the catabolic diversity index (Shannon functional diversity index, H) were computed together with the Shannon Evenness (E) index (H) calculated by the free software Past 3.14 for environmental data.

The absorbance percentages of each substrate were determined according to Sala et al. [42] and 2% absorbance of the total absorbance per plate was used as a threshold for substrate utilization.

The substrata were divided in six guilds of carbon substrates: complex carbon sources (polymers), carbohydrates, phosphate carbon sources, carboxylic and acetic acids, amino acids and amines.

#### 2.5.2. Potential Rates of Enzymatic Activities

Microbial enzymatic activities on proteinaceous (leucine-aminopeptidase, LAP) and glucidic (ß-glucosidase, ß-GLU) organic matter, and on organic phosphates (alkaline-phosphatase, AP) were estimated on whole brine samples in order to quantify both particulate and dissolved enzymes. Enzyme assays were performed using the Hoppe’s method, which suggests the use of specific fluorogenic substrates [43] for LAP, ß-GLU and AP measurements. Particularly, brine samples were suspended in sterile distilled water at a 1:10 (*w*/*v*) ratio and vortexed. The supernatant obtained after sedimentation was used as the enzymatic extract for the subsequent assays. Increasing amounts of each substrate were added to triplicate 5-mL sub-volumes of each sample and the fluorescence released by substrate hydrolysis was measured with a Turner TD-700 model fluorimeter, attime 0 (initial time) and 3 h after incubation at 5 °C. Calibration curves with the standards 7-amino-4-methylcoumarin (MCA) or 4-methylumbelliferone (MUF) were performed for LAP or for β-GLU and AP, respectively. Results were elaborated and expressed as described in Papale et al. [44] and references therein [45,46,47]. Cell-specific enzymatic activity was determined by normalizing the total (bulk) enzymatic activity to the prokaryotic cell abundance, taking into account the initial dilution of the brine samples. 

### 2.6. Prokaryotic Community Diversity and Composition

The prokaryotic community diversity and composition were evaluated by Ion Torrent sequencing, as it is described in the Section 2.6.1, Section 2.6.2, Section 2.6.3 and Section 2.6.4, respectively.

#### 2.6.1. DNA Extraction

Brine sub-samples (between 150 and 600 mL) were subjected to filtration (0.22 μm-pore size polycarbonate filters, diameter 47 mm, Millipore). Membranes were stored at −20 °C until analyses. The PowerSoil DNA extraction kit (MoBio Laboratories, Carlsbad, CA, USA) was used for DNA extraction in duplicate from membranes. DNA concentrations and purity were quantified by using a NanoDrop ND-1000 UV-vis Spectrophotometer (NanoDrop Technologies, Wilmington, DE, USA).

#### 2.6.2. Amplification of 16S rRNA Genes and Ion Torrent Sequencing

The extracted DNAs were used to amplify the V1-V2 region of 16S rRNA gene of Bacteria (primers 27f 5′-AGAGTTTGATCCTGGCTCAG-3′ and 338 5′-GCT GCC TCC CGT AGG AGT -3′) and the most conserved V3-V4 region of Archaea (primers ARC344f 5′-ACGGGGYGCAGCAGGCGCGA-3′ and 693r 5′-GGATTACARGATTTC-3′). Two PCR amplifications (in duplicate) were performed according to Papale et al. [44]. Briefly, in order to reduce biases in massive sequencing, a two-step PCR protocol was applied: the first step consisted of a conventional PCR, and then amplicons were used as templates in the second PCR with barcoded primers for Ion Torrent sequencing. PCR products were purified, quantified, pooled for emulsion PCR and sequenced as described in Papale et al. [44]. The Ion Torrent PGM platform (Life Technologies, USA) with Hi-Q sequencing chemistry was used to reduce the problem of poor homopolymer sequencing [48,49]. All steps during amplification and sequencing were checked using negative controls.

#### 2.6.3. Post-Run Analysis

To assess the sequencing error type and rate in concert with the read quality score, the FastQC tool was adopted to analyze the reads. This tool simplifies executing quality control (QC) on FastQ files by aggregating QC data like quality by position, sequence content, GC content, and adapter content. The raw data were analyzed as described in Papale et al. [44] and references therein [50,51,52] using the bioinformatics analysis software MOTHUR (version 1.39.5). All quality checked sequences were taxonomically classified using the reference alignment with 100 iterations and a minimum bootstrap confidence score of 80%, and clustered into (OTUs) at a 97% similarity level. Consensus sequences of each OTU were again classified with a minimum consensus confidence threshold of 80% [52].

Ion Torrent sequence data obtained from this study have been registered as NCBI Bioproject PRJNA435985.

#### 2.6.4. Analyses of Prokaryotic Communities 

The taxonomic profiles were generated by classifying the sequences against the Silva database [53], and the distance matrices (label 0.03) were created to obtain the OTU table for subsequent analyses. Starting from these data, the matrix and the rarefaction curves for both brines were calculated for a 0.03 distance cutoff [54].

Obtained OTUs for Bacteria and Archaea by 16S rRNA gene amplicon sequencing (reads were normalized to the lowest number of read by the Mothur sub.sample command) were used to generate Venn diagrams by using the R software version 3.0.1, VennDiagram package [55]. 

Heatmaps were constructed to display the presence and abundance of bacterial and archaeal genera in the brine samples, based on Bray–Curtis dissimilarities. The analysis was performed by using Heatplus and Gplots packages in an R environment (R 3.4.4). The genera whose relative abundance was less than 1% were eliminated.

Diversity indices calculations were performed after subsampling based on the lowest number of reads. Alpha diversity measures were evaluated by Mothur software, including the terminal richness estimation (Chao1). Mean value and differences between diversity indices were calculated and assessed for significance using one-way ANOVA (Excel software).

## 3. Results

### 3.1. Microbial Cell Abundances

#### 3.1.1. Virus-Like Particle and Prokaryotic Cell Abundances by Flow Cytometry (FC)

The virus-like particle (VLP) abundances were in the order of 10^10^ VLP L^−1^ and higher counts were obtained in TF4 (3.47 ± 0.18 × 10^10^ VLP L^−1^) than TF5 (2.57 ± 0.08 × 10^10^ VLP L^−1^). Different VLP subgroups (namely V1, V2 and V3) were detected. The fraction of VLP detected in both samples was mainly represented by smaller particles in V1 sub-group (73% and 80%, in TF4 and TF5, respectively). Lower percentages were detected for the V2 sub-group (27% and 19%) and were negligible for the V3 one (<1%).

Prokaryotic abundance (PA^C^) in both samples was in the order of 10^9^ cells L^−1^, but in TF5 it was two times higher than in TF4 (mean values 1.80 ± 0.09 and 3.77 ± 0.13 × 10^9^ cells L^−1^ in TF4 and TF5, respectively). The viral to prokaryotic abundances ratios (VPR)—determined by FC—were 19 and seven in TF4 and TF5, respectively, and VPR was 2.7 times higher in TF4 than in TF5.

Prokaryotic cells with a high nucleic acid (HNA) content were always higher than LNA, accounting for the 54% and 62% in TF4 and TF5, respectively.

#### 3.1.2. Prokaryotic Cell Abundance, Morphometry, Morphology and Biomass by Image Analysis (IA)

Similarly to PA^C^ determinations, total prokaryotic cell abundances (PA) estimated by IA were in the order of 10^9^ cells L^−1^ in both samples, with a higher value in TF5 (mean value 8.10 ± 0.71 × 10^9^ cells L^−1^) than in TF4 (5.01 ± 0.23 × 10^9^ cells L^−1^). The morphometry and morphologies of the prokaryotic cells in the studied samples are reported in Table 1.

Cell lengths varied between 0.21 and 3.01 µm and widths between 0.21 and 0.95 µm. On the whole, VOL ranged between 0.005 and 0.492 µm^3^ with a mean value of 0.090 ± 0.097 µm^3^ (data not shown), with wide differences between the two different brines. In fact, in TF4, very small cells were detected (<0.06 µm^3^) with an averaged VOL of 0.040 ± 0.027 µm^3^, while in TF5 larger cell sizes were observed (averaged VOL 0.105 ± 0.104 µm^3^). The high standard deviation in TF5 was related to the occurrence of cells having different sizes. According to their shape, the prokaryotic cells were classified into four distinct morphotypes: coccobacilli, cocci, rods and curved rods. In TF4, curved rods were absent, cocci were the main morphotype, contributing to the 44% of the total prokaryotic cells, followed by rods (30%) and coccobacilli (26%) (data not shown). Contrastingly, in TF5, cocci accounted for the 28%, rods and coccobacilli for 33% and curved rods for the 5% of the total prokaryotic cells. The large cell sizes observed in TF5 were mainly due to the curved rods which—although few in number—were the biggest ones (data not shown). 

The prokaryotic biomass varied between the two brines, and was 3.6 times higher in TF5 (242 µg C L^−1^) than in TF4 (67 µg C L^−1^).

### 3.2. Evaluation of Viable (Live/Dead) and Respiring Prokaryotic Cells (CTC+)

Highest percentage of live cells (67%) was observed in TF4, while the highest percentage of dead cells was in TF5 (59%). Differently, respiring cells (CTC+) were in the order of 10^8^ cells L^−1^ in TF4 and 10^9^ cells L^−1^ in TF5, and represented 18% and 30% of total counts obtained by DAPI staining, respectively (Supplementary Figure S1).

### 3.3. Metabolic Potential and Enzymatic Activities

#### 3.3.1. Physiological Profiles

Concerning the Biolog-Ecoplate, for raw substrate utilizations recorded at the plate level (ASCD), different results were found between the brines (Table 2a). 

Low levels of ASCD resulted in TF4, showing a mean value of 0.057 ± 0.018, with a maximum of 0.075 in 21/11/2014. The highest number of positive wells (11) was detected in the period from 03/12/2014 to 09/12/2014, amounting for the 16% of the total number of wells. In TF5, the ASCD mean value was 0.126 ± 0.154 and the maximum (0.542) was reached the last day of readings (11/12/2014), when the 100% of the total wells resulted in absorbance over 0.10 OD. In Table 2b, the values of biodiversity indices for the two brines are presented. They were calculated when maximum ASCD values were reached; namely, after 4 days from inoculation for TF4 and after 24 days for TF5. Higher values of physiological diversity (H), substrate richness (S, positive wells) and substrate evenness (e^H/S^) and equitability (J) were determined for TF5 than for TF4.

**Figure 2:**
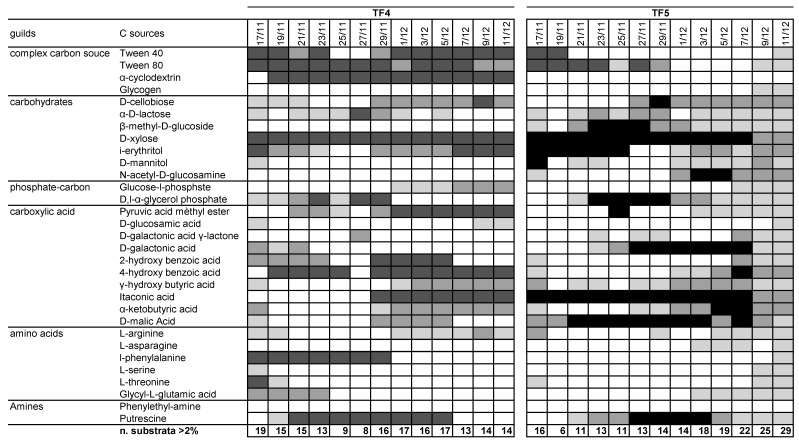
Pattern of utilization of the 31 carbon sources for TF4 and TF5 samples. The box color is indicative of the percentage absorbance range of the total absorbance of the plate. Values are as follows: white < 2%; light grey 2–4%; dark grey 4–6%; black > 6%. Below each column, the number of substrates with greater or equal to 2%, 4% and 6% absorbance for each sample.

The patterns of utilization of the 31 carbon sources with percentage of absorbance >2% were summarized in Figure 2 Notwithstanding the differences in ASCD and S, the number of substrates was slightly lower in TF4 than in TF5 (mean values 14.3 ± 3.1 and 16.2 ± 6.2, respectively). However, the number of substrates with percentages of absorbance >2% in TF4 was fairly constant over the entire reading time (R^2^ = 0.0231; y = 0.022x + 936.77), while in TF5 the number increased with time, totaling 29 on the last day of experiment (R^2^ = 0.72; y = 0.6703x + 28119). Considering the threshold of >2%, glycogen, β-methyl-D-glucoside, N-acetyl-D-glucosamine, L-asparagine were not metabolized in TF4; α-cyclodextrin and phenylethyl-amine were not in TF5. Considering the threshold at >6%, Tween 40, Tween 80, D-xylose, itaconic acid and putrescine were highly utilized by both brine samples; α-cyclodextrin, 4-hydroxy benzoic acid and l-phenylalanine in TF4 only; i-erythritol, D-galactonic acid and D-malic Acid were utilized only in TF5. 

**Figure 3:**
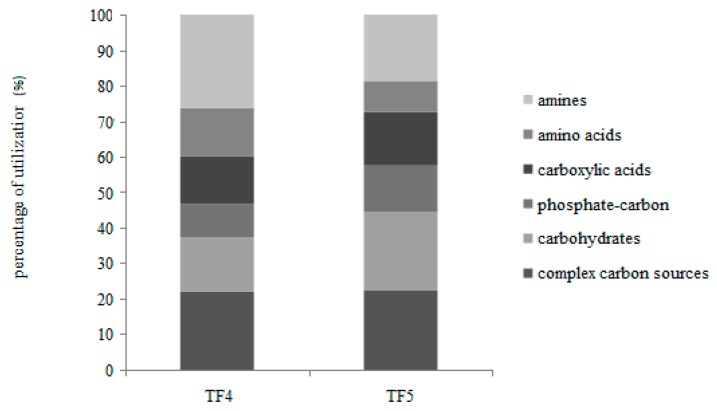
Individual carbon source oxidation—as a percentage of the total absorbance—in TF4 and TF5 brines.

On the whole, the percentages of carbon source utilization showed that the polymers and amines were the most utilized substrates, followed by carbohydrates and carboxylic acids (Figure 3).

Different utilization patterns of the six guilds were obtained between TF4 and TF5. In particular, with the exception of the complex carbon sources (polymers) that were similarly utilized, the amines and amino acids were preferentially metabolized in TF4, while the carbohydrates, phosphate-carbon sources and carboxylic acids were in TF5.

#### 3.3.2. Enzymatic Activities

The results are reported in Table 3, where the bulk activity rates (expressed as V_max_) of the enzymatic hydrolysis, the cell-specific enzymatic activities and the reciprocal molar ratios of the enzymatic activities are shown.

Mean bulk LAP activity rates ± standard deviation (*n*= 3 replicates) ranged from 2.04 ± 0.72 to 1.97 ± 0.25 nmol L^−1^ h^−1^ in TF4 and TF5, respectively. Additionally, mean bulk AP activity rates were quite similar between the two samples (1.05 ± 1.26 and 1.03 ± 0.09 nmol L^−1^ h^−1^ in TF4 and TF5, respectively). Significant differences (*p* <0.01) were, however, found for bulk β-GLU activity, which was negligible in TF5 (0.12 ± 0.09 nmol L^−1^ h^−1^) and remarkable in TF4 (2.53 ± 3.90 nmol L^−1^ h^−1^). At the single cell level, the patterns of cell specific enzymatic activities reflected those of bulk V_max_ values, showing cell-specific glycolytic and phosphatase activities that all dominated in TF4. Significantly higher (*p* <0.01) ratios of C-acquiring to P-acquiring enzymes and C-acquiring to N-acquiring enzymes were measured in TF4 than in TF5. No differences between the two brine samples were found with respect to the reciprocal ratios of N-acquiring to P-acquiring enzymes.

### 3.4. Prokaryotic Community Diversity

#### 3.4.1. The 16S rRNA Gene Amplicon Sequencing

The total number of bacterial reads were 31,863 and 32,823 for TF4 and TF5, respectively. Those of Archaea were 36,555 and 29,464 for TF4 and TF5, respectively. Following the trimming step, the number of high-quality reads were 18,322 and 18,394 for Bacteria and 13,425 and 11,713 for Archaea for TF4 and TF5, respectively. Overall, the bacterial and archaeal reads were resolved in a total of 691 (414 in TF4 and 430 in TF5; 153 OTUs were common to both samples) and 1145 OTUs (659 in TF4 and 696 in TF5; 210 OTUs were common to both samples), respectively. OTUs were equally distributed between samples (37.8% and 40.1% for Bacteria, and 39.2% and 42.4% for Archaea in TF4 and TF5, respectively). Brines from TF4 and TF5 shared only 22.1% and 18.3% of total bacterial and archaeal OTUs respectively (Supplementary Figure S2). To analyze both communities, the percentages related to phyla and genera in bacterial and archaeal populations were referred separately to the total bacterial and archaeal read numbers, respectively.

A relatively high alpha-diversity was found for Bacteria and Archaea, and the values were quite similar between the brines. Specifically, Archaea showed higher values of Shannon H index, with the highest value recorded in TF5 (6.863). Instead, Chao1 diversity estimator values indicated that the diversity was not well covered for the prokaryotic communities.

##### Bacteria

**Figure 4:**
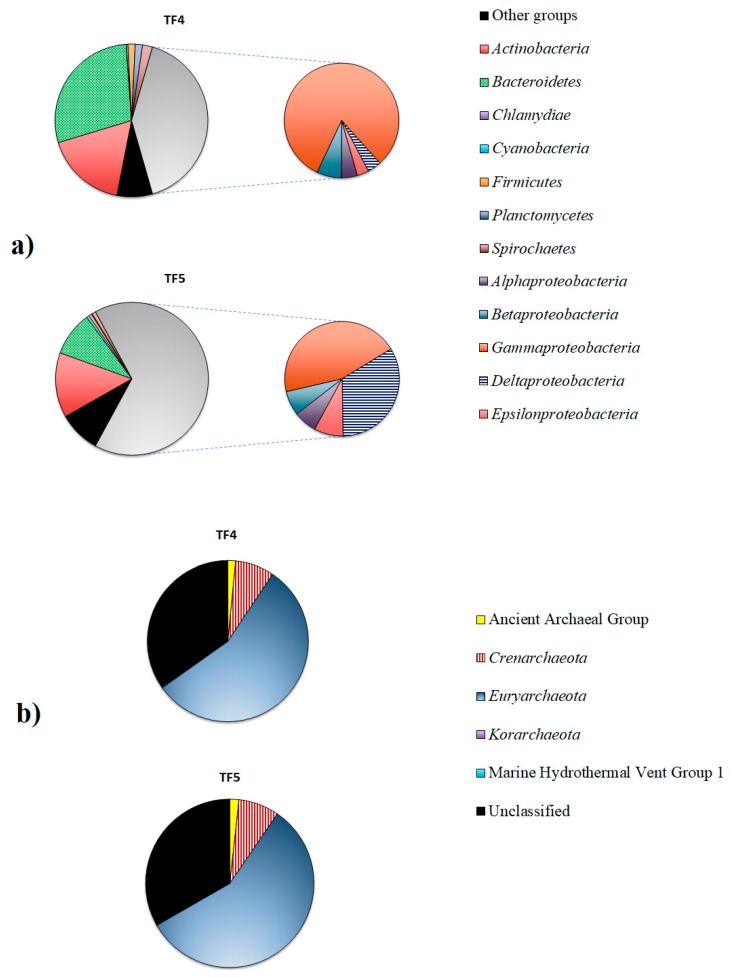
Bacterial (**a**) and archaeal (**b**) communities in TF4 and TF5 brines.

Proteobacteria dominated in both samples, but were higher in TF5 (65.9%) than in TF4 (41.1%). The second most abundant phylum was Bacteroidetes, that was predominant in TF4 (28.6%), rather than in TF5 (9.4%), followed by Actinobacteria (17.2% and 13.7% in TF4 and TF5, respectively). Spirochaetes (2.1% and 0.8% in TF4 and TF5, respectively), Firmicutes (1.5% and 0.6% in TF4 and TF5, respectively) and Planctomycetes (1.6% and 0.1% in TF4 and TF5, respectively) were less represented in both samples. The relative abundances of the remaining phyla (i.e., Acidobacteria, Aquificae, Chloroflexi, Deinococcus-Thermus, Gemmatimonadates, Lentisphaerae and Verrucomicrobia) were less than ≤1% each, and they were grouped into the “Other groups” (7.6% and 8.9% in TF4 and TF5, respectively) (Figure 4a). 

The composition of Proteobacteria in the two brine samples clearly differed at class level. Gammaproteobacteria were dominant in both samples, but were more abundant in TF4 (33.4%) than in TF5 (29.6%). Deltaproteobacteria (22.0%) was the second most abundant proteobacterial class in TF5, while they were less represented in TF4 (1.6%). Sequences related to Epsilonproteobacteria (1.4% and 5.4% in TF4 and TF5, respectively), Betaproteobacteria (2.9% and 4.6% in TF4 and TF5, respectively) and Alphaproteobacteria (1.8% and 4.3% in TF4 and TF5, respectively) were more abundant in TF5 than in TF4.

**Figure 5:**
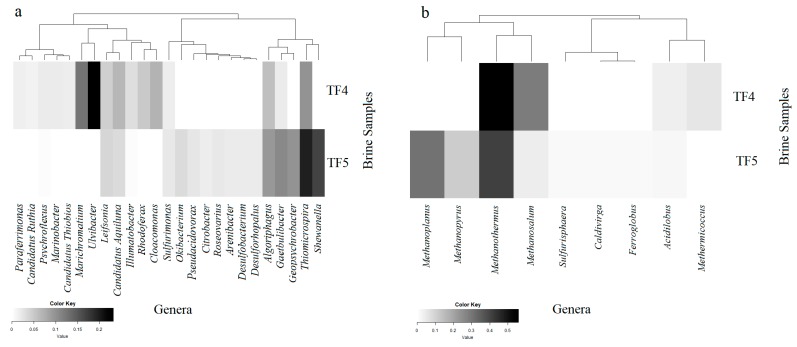
Heatmaps of presence and abundance of bacterial (**a**) and archaeal (**b**) genera in the brine samples. Color blocks represent the relative abundance of genera. More darkness indicates a higher relative abundance.

Of the total high-quality bacterial sequences obtained from TF4 and TF5, about 14% and 18%, respectively, were not classified at the genus level. A total of 268 and 269 genera from TF4 (from 0.01% to 18.3% of total sequences) and TF5 (from 0.01% to 10.9%), respectively, were resolved from the rest. Only genera occurring at ≥0.1% of the total bacterial sequences, resulting in 53 and 41 genera from TF4 and TF5, respectively, are reported in Figure 5a and Supplementary Table S1.

In line with the marked dominance of Bacteroidetes in TF4, most of classified sequences at the genus level were affiliated with this subphylum. The dominant Bacteroidetes genus in TF4 was *Ulvibacter* (18.3% of total sequences), followed in abundance by *Gaetulibacter*, *Psychroflexus* and *Algoriphagus*, whereas *Algoriphagus* dominated in TF5.

Within the Gammaprotobacteria, *Marichromatium* (9.9%), *Thiomicrospira* (7.8%), and *Marinobacter* (1.6%) represented the dominant genera in TF4, whereas *Thiomicrospira* (10.8%) and *Shewanella* (9.3%) dominated in TF5. 

Differently to TF4, sequences within Deltaproteobacteria were more abundant in TF5 brine and were mostly related to *Geopsychrobacter* (5.5%), followed by *Desulfobacterium* and *Desulforhopalus*. The dominant epsilonproteobacterial genus retrieved in both brines was *Sulfurimonas*, which was more abundant in TF5 rather than in TF4.

The most abundant genera within Betaproteobacteria were *Rhodoferax* (3.8%) in TF4 and *Pseudacidovorax* (1.3%) in TF5. More numerous genera within Alphaproteobacteria were retrieved from TF5 than from TF4 and sequences related to the genus *Roseovarius* were more abundant in TF5. 

Sequences affiliated with Actinobacteria were mainly related to the genus candidates *Aquiluna* (followed by *Illumatobacter* and *Leifsonia)* in TF4. Among Spirochaetes, the dominant genus was *Cloacamonas* in TF4.

##### Archaea

The archaeal communities were mainly constituted by Euryarchaeota (55.8% and 57.1% in TF4 and TF5, respectively), followed by Crenarchaeota (about 7.8% in both samples) and Ancient Archaeal Group (1.6% and 1.8% in TF4 and TF5, respectively). Korarchaeota (0.1% and 0.02% in TF4 and TF5, respectively) and the Marine Hydrothermal Vent Group1 (0.2% and 0.1% in TF4 and TF5, respectively) were less represented. A total of 34.6% and 33.1% of reads were unclassified in TF4 and TF5, respectively (Figure 3b). 

Of the total high-quality archaeal sequences from brine, about 64% from TF4 and 56% from TF5 were not classified at the genus level. A total of 16 and 22 genera were resolved from TF4 (from 0.01% to 19.1% of total sequences) and TF5 (from 0.01% to 17.4% of total sequences), respectively. Only genera occurring at ≥0.1% of the total archaeal sequences are reported in Figure 4b and Supplementary Table S2. 

Retrieved crenarchaeotal genera were mainly related to hyperthermophiles. Among them, *Acidolobus* (dominant in TF4), *Aeropyrum* and *Ignicoccus* were retrieved in both TF4 and TF5. Most euryarchaeotal sequences were related to methanogens. *Methanothermus* represented the dominant genus in both brines, followed in abundance by *Methanosalsum* and *Methermicoccus* in TF4, but by *Methanoplanus* and *Methanopyrus* in TF5.

Among less abundant euryarcheotic genera, members of Halobacteria related to the genus *Halorhabdus* were common to both brines, whereas genera *Haloarcula* and *Halobaculum* were detected only from TF4, and *Halobacterium* only from TF5. The hyperthermophilic genus *Thermococcus* was retrieved in both samples, whereas *Ferroglobus* and *Picrophylus* were only in TF4.

## 4. Discussion

Polar habitats harbor diverse and metabolically active microbial communities [17,56,57,58]. To the best of our knowledge, this is the first comprehensive study which includes quali-quantitative and metabolic analyses of microbial communities inhabiting hypersaline brines from a perennially ice-covered lake in the Tarn Flat area. Recently, Chua et al. [58] defined the Antarctic briny environments as liquid oases for microbial life. Hypersaline brine lenses are unique aquatic systems that are thought not to undergo changes via external influence over geological timescales [59]. In this regard, the brine TF4 had probably experienced a chemico-physical exchange with the atmosphere, whereas TF5 was supposed to be isolated since 12000 cal years BP [23]. A combined analytical approach, which included the estimation of microbial abundances (by means of microscopic image analysis and flow cytometry), and metabolic activities (by means of carbon substrate utilization profiles through Biolog-Ecoplate and enzyme activity measurements), and the analysis of the prokaryotic community diversity and composition (by a Ion Torrent approach), was applied in this study. 

### 4.1. Microbial Abundances

Prokaryotic abundances were comparable to those found in the Antarctic Lake Vida located in the McMurdo Dry Valleys [60], and higher than those reported for the Lake Vostok [61] and Lake Bonney [56]. High abundances of viral particles were recorded in the two brines, confirming previous determinations in other cold extreme environments and in Antarctic lakes ([62] and references therein). The high viral densities suggest viruses have an active and dynamic role in the microbially dominated polar systems [56,63], such as perennially ice-covered lakes. Moreover, their important role in genetic exchange through lysogeny might be considered [62]. The abundance of environmental viruses could shape the microbial community, since microbe–virus interactions play a significant role in microbial mortality and ecosystem functioning [64,65,66]. Viral influences differ according to the ecosystem, especially in highly productive ones. With anincrease of the prokaryotic host population, factors such as the emergence of resistant strains and host-virus specificity may influence the microbial community, principally the prokaryotic community [56,67]. Because of that, to understand viral abundance we need to consider microbial abundance [68,69]. For such purposes, researchers have observed the virus-prokaryotic cell rate (VPR) as a statistical proxy for aquatic environments [69,70,71,72,73,74,75]. It is a consensus-view that VLP abundance exceeds the prokaryotic abundance by one order of magnitude [72,73]. However, systematic observation of this relationship in different studies showed substantial variations in this relationship, depending on the type of aquatic ecosystem [67,69,75]. The interaction between bacteriophages and their hosts is explained by the virus to prokaryotic ratio (VPR) [62,63]. The 2.7 times higher VPR value in TF4 than in TF5 might be explained by the specificity of the virus-host relationship, along with the reduction of burst size, or the development of immunity by hosts; or by the change in life strategy of viral particles for lysogeny [49,66,75,76,77]. In Baltic sea-ice samples, a distinct phage-host system was characterized relatively to the psychrophilic bacterium *Shewanella* [78], which was isolated in TF5 only. 

The existence of different microbial active sub-groups, with a high percentage of HNA cells at TF5 (Supplementary Table S1) could be explained by the inherent characteristics of the TF5 sample, which was thicker, saltier and had more microbial biomass than TF4. The HNA bacterial groups are more versatile bacteria with large and flexible genomes, while LNA groups show less adaptability have and smaller genomes [79]. A recent study showed that the HNA bacteria were responsible for a richness increase in a marine sample [79]. Microbial adaptation to extreme conditions may also involve changes in cell morphology. The observed microbial communities revealed small cell volumes (mean total value of 0.089 ± 0.094 µm^3^), higher than those reported by Mosier et al. [60] for the Lake Vida. However, significant differences (one-way ANOVA: *p* <0.005) in prokaryotic cell volume between the two brines occurred, with larger cell volumes in TF5. Cell sizes smaller than 0.1 µm^3^ have often been related to stressed, starved, or dormant cells in extremely cold and salty environments [19,80]. Combining results from microscopic and flow cytometric determinations, different microbial communities in the two pockets were recognized in terms of prokaryotic cell abundances, HNA percentages, respiring cells, morphometrics and biomass. Differently, the discrepancy provided by L/D to CTC+ estimates could evidence the existence of actively growing cells [81] more abundant in TF5 than in TF4. A possible explanation for this finding could be the probable presence of detritus particles in TF5, since attached microorganisms are proportionally more active than free living ones [81].

### 4.2. Metabolic Potential and Enzymatic Activity

The potential metabolic functions and roles played by the microbial communities within the two Antarctic brines were evaluated by Biolog-Ecoplate assay and the determination of enzymatic activity rates. The raw metabolic responses at community level, such as those obtained by Biolog-Ecoplate profiles, were low in both brines, in a range near the minimum detectable. This scarce activity might be attributable to the environmental constraints, mainly the extremely cold temperatures and brine isolation from nutrient supply byallochthonous inputs, with a consequent inhibition of community functionality [82,83]. Nevertheless, the amount of reduced substrates indicated extensive metabolic potentials, especially in TF5, where all the compounds were utilized. In terms of potential functionality (expressed as the percentage of guild’s utilization), the complex carbon sources were well metabolized, mainly in TF4, and these findings might be related to their easy degradability and antifreeze characteristics [84]. Interestingly, TF4 was characterized by a high number of sequences related to Bacteroidetes with specialized roles in the natural carbon cycle by hydrolyzing macromolecules [85,86,87,88], probably through the production of extracellular enzymes. Since in polar environments, microbial survival depends on the ability of microorganisms to prevent the growth of ice crystals inside their cells, freeze-tolerant strategies allow the inhibition of ice crystal growth [38,89]. 

Diverse metabolic responses were observed for the other carbon sources: amines and carbohydrates were the most exploited compounds in TF4 and TF5, respectively, whilst phosphate-carbon sources and aminoacids were the lowest ones. Previous data obtained in other frozen matrices, i.e., Arctic permafrost soils [84,90] and Antarctic active and deep permafrost layers [39,47], showed that polymeric compounds and carbohydrates were potentially well utilized. The latest data shows constitutional, functional biomolecules (acting both for energy storage and as structural components) in all living organisms, and common metabolites in almost all microbial habitats. Nevertheless, carbohydrates were well utilized in TF5 only. Differently, metabolic responses versus nitrogen compounds, i.e., putrescine, were high in TF4, coherently with Laybourn-Parry and Pearce’s [62] statements on the distribution of nitrogen compounds and the role of nitrifying bacteria and nitrogen fixation in Antarctic lakes and ponds. At the same time, aminoacids were used at negligible level in TF5, in accordance with La Ferla et al. [39] and Wagner et al. [90] in other Arctic and Antarctic matrices; namely, permafrost. Hence, different pathways of metabolic potentials were found in TF4 and TF5, asserting the metabolic diversity among the microbial communities living in each brine.

As determined by the enzymatic hydrolysis assays, the metabolic activities potentially played by the microbial communities in the decomposition of high molecular weight organic substrates were different between the two analyzed brine samples. While bulk LAP and AP showed quite consistent levels of activity, the different bulk β-GLU activity rates measured between the two brines suggested that the samples differed for the quality of their organic substrates. The bulk enzymatic activity rates were in the order of β-GLU > LAP > AP in TF4, while in TF5, enzymes dominated in the order LAP > AP > β-GLU. The reciprocal magnitude order of enzymes may vary depending on several factors, among which, the geographical latitude, season and quality of the available organic matter play major roles [91,92]. In marine environments, proteins are often metabolized faster than polysaccharides, as indicated by LAP to β-GLU ratios being>1 [43,93,94]. Conversely, in low salinity environments high levels of β-GLUare expressed [92]. In both the samples, higher values of bulk LAP compared to bulk AP indicated a greater ability of the microbial communities to decompose proteins rather than organic phosphates. The peak of bulk β-GLU recorded in the TF4 sample indicated an active metabolism of polysaccharides compared to the TF5 sample. This could represent an adaptative strategy to front the nutrient paucity [95]. In Antarctic brines, extracellular enzymatic activity has never been investigated before and this study is a first contribution to this interesting research topic. The detection of enzyme activities in brine samples suggests physiological adaptations of microorganisms under severe, low temperature and high salinity, environmental conditions [96,97,98]. Cold-adapted and salt-tolerant microbial proteolytic, glycolytic and phosphatasic enzymes were detected in Arctic sea ice from the Canada basin [99]. Although not all prokaryotic cells may necessarily be active, and the contribution of organisms other than prokaryotes is unknown, cell specific enzyme activities, as obtained by scaling enzyme activity rates to the cell abundance, can give new insights on degradation capability and metabolic potential at a single cell level [43,100]. In TF brines, cell specific activities showed high values of both LAP and β-GLU in TF4. 

Considering the reciprocal enzyme molar ratios, as an indicator of metabolic activities directed towards organic N (i.e., LAP) and P (i.e., AP) uptake with respect to C (i.e., β-GLU) [45,46], in the examined TF brines’ high C:P and C:N ratios suggested that carbon was preferentially mobilized compared to P and N. Such a finding, related to high β-GLU in the TF4 sample, confirmed that shifts in enzyme ratios can be also associated to changes in element-use efficiency [101]. The microbial communities inhabiting the TF brine samples were able to decompose proteins and organic phosphates with similar efficiency, as suggested by the comparable ratios of N-acquiring to P-acquiring enzymes.

### 4.3. Prokaryotic Community Diversity and Composition 

The application of culture-independent methods allowed obtaining complementary information on the diversity and composition of the prokaryotic communities inhabiting TF lake brines. 

Overall, similarities at both phylum and genus levels were found with data previously obtained for brines from other Antarctic areas [5,17]. TF4 and TF5 brines shared members mostly affiliated with the same taxonomic groups, but the differences encountered in their relative abundances resulted in distinct bacterial and archaeal assemblages. Bacteroidetes (mainly represented by the genus *Ulvibacter*) and Gammaproteobacteria (with the predominance of the genus *Thiomicrospira*) were more abundant in TF4, whereas Deltaproteobacteria, mainly represented by the versatile sulphate-reducing bacteria (SRB), were predominant in TF5. 

Due to the high salinity content in the TF brines, numerous sequences were affiliated to halotolerant and moderately halophilic microorganisms, both aerobes and anaerobes, belonging to the bacterial and/or archaeal domains e.g., [102,103]. The TF brines hosted isolates belonging to the genus *Psychrobacter*(within Gammaproteobacteria) and were able to grow up to 17–19% in NaCl (data not shown). That genus, in addition to *Marinobacter*, appears to be cosmopolitan in cold Antarctic brines [5,17,19,58,104] and hypersaline Antarctic lakes [105]. Halophilic species have been previously retrieved in Antarctica in samples of geothermal and saline soils [106]. The occurrence of some bacterial species (e.g., *Marinobacter*, *Polaromonas*, *Ulvibacter*) that are commonly observed in seawater may derive from the possible occurrence in the lake bottom of an ancient ice remnant from the Ross Ice Shelf (as shown in Figure 3a,b of Forte et al. [23]), which in according with Anderson et al. [107], should be still grounded in this sector of Victoria Land at approximately 12 kyr BP.

The different geothermal sites provide optimal habitats for the growth of thermophilic and hyperthermophilic microorganisms in Antarctica e.g., [106,108,109,110,111], and the natural barriers that separate them make this continent an ideal model to study how thermophiles evolve in separated Antarctic locations. Indeed, despite the fact that Antarctica is considered a cold environment, geothermal activity has been reported for both Antarctic circumpolar islands (e.g., South Sandwich Islands and Deception Island) and geographical sites on the continent (e.g., Mount Erebus, Mount Melbourne and Mount Rittmann). Moreover, 91 volcanoes have recently been discovered in a massive region known as the West Antarctic Rift System [112]. In this study, for members assigned to thermophilic bacteria (Aquificae and Deinococcus/Thermus), hyperthermophilic Crenarchaeota (*Acidianus*, *Aeropyrum*, *Ignicoccus* and *Sulfolobus*) and Euryarchaeota (*Methanothermus*, *Methanopyrus* and *Thermococcus*) were common to both brines, indicating that they host the mostthermally tolerant Archaea. The observed increaseof crenarchaeotalphylotypes with depth, as well as the fact that their sequences were mainly related to well-known hyperthermophiles of deep-sea hydrothermal vent origin, could be supported by the hypothesis put forward by Forte et al. [23] of a possible deep circulation of saline brine. Indeed, this finding would be surprising as no geothermal activity has been reported in the Tarn Flat area, even in the past, but as a speculation it is possible that thermophilic and hyperthermophilic microorganisms, or their DNA, may derive from this deep circulation. 

The abundance of sequences related to sulphate-reducing bacteria (SRB), e.g., *Geopsychrobacter* and *Desulforhopalus*, and methane-generating archaeal groups within Methanobacteriales (17.2%), Methanomicrobiales (15.4%) and Methanosarcinales (0.16%), likely reflects the presence of a syntrophic consortium, cycling carbon compounds in anaerobic conditions, in the confined TF5 brine. These findings may explain the abundant gas bubbling observed during the drilling, only withthe TF5 brine. TF5 probably experienced anoxic conditions or derived from the upward movement of saline brine from a sub-surface anoxic system filling the TF5 lake basin. As it was observed by Hinrichs et al. [113] for marine sediments, certain Archaea reverse the process of methanogenesis by interaction with SRB. Members of versatile SRB include species that oxidize organic compounds (including acetate) to CO_2_, and several species that can grow autotrophically with CO_2_, H_2_ and SO_4_^2-^. A microenvironment characterized by a strong deficiency in hydrogen, acetate or other possible intermediates representconditions that favorthe methane anaerobic oxidation via reversed methanogenesis in the consortia [114]. 

The occurrence of methanogens has been previously investigated by Stibal et al. [57] in Arctic and Antarctic subglacial sediments, and reported for a number of systems (e.g., deep ocean, permafrost, lake sediments) characterized by a combination of anoxic conditions, a suitable carbon substrate and the absence of high energy yielding electron acceptors, with all of these factors creating favourable conditions for methanogenic Archaea, and hence methanogenesis [57]. Members of Methanosarcinales include metabolically diverse methanogens, since they may form CH_4_ from several simple methyl-group-containing compounds (acetate, methanol, methylamines and methyl sulphide) [115]. Abundant sequences retrieved only in TF5 brine were related to Methanopyrales, and were actually represented by the only hyperthermophilic species, *Methanopyruskandleri*, able to produce methane by CO_2_ reduction with H_2_. In contrast to other methanogens from non-brines’terrestrial habitats, Antarctic methanogens could possess natural adaptation mechanisms (including protein structural changes, efficient DNA repair mechanisms and starvation) to effectively counterthe extreme conditions of brines (such as subzero temperatures and increased salinity). According to other authors [116], due to the unique properties, methanogenic Archaea from TF brines should be considered as primary candidates for possible extraterrestrial life in Martian sub-surfaces. Additionally, halophiles, thanks to their ability to cope under extremes of temperatures and salt concentrations, could be true candidates for inhabiting Martian environments [117]. Recently, Oren et al. [118] suggested that halophilic life, mainly consisting of salt-requiring or salt-tolerant microorganisms similar to the halophiles on Earth, may exist in liquid brines on Mars, growing anaerobically by reduction of perchlorate and chlorate.

Even though the two brine pockets were separated by a thin ice layer, sulphide production by SRB in the TF5 brine may explain the occurrence of sulphur-oxidizing bacterial (i.e., *Rhodoferax*, *Sulfurimonas* and *Thiomicrospira*) and archaeal members (i.e., *Acidianus* and *Sulfolobus*) in the upper TF4 brine. Interestingly, the psychrophilic marine autotrophic sulfur oxidizer *Thiomicrospiraarctica* also dominated in the Blood Falls, the surface manifestation of brines released from below the Taylor Glacier (McMurdo Dry Valley, Antarctica) [17]. The occurrence of this obligate chemoautotroph could imply an in situ primary production during the Antarctic winter, in the total absence of sunlight and at permanently low temperatures [10,119].

## 5. Conclusions

The most likely hypotheses to explain the microbial community differentiation recorded in the examined brines could be the following: (i) the different chemical and physical composition of the brines; (ii) the presence of a barrier represented by a thin layer of ice; and (iii) the different geological origins of the brines. Indeed, the results of this study have distinct implications, firstly linked to the geological origin of the brines and secondly to the main characteristics of the confined environments that were analyzed. Interestingly, the abundance of Bacteria and Archaea related to chemoautotrophic phylotypes was consistent with the oxidation of reduced sulfur and nitrogen compounds. This process plays an important role as a pathway for primary production in this peculiar ecosystem, gaining energy efficiently at extremely low temperatures and in the absence of sunlight (e.g., during the dark Antarctic winter). Further, the detection of methanogens supported the hypothesis that methane cycling occurs in the analyzed lake, and suggested that the Tarn Flat brine system can be independently sustained by microbially-mediated carbon fluxes following in situ CO_2_ fixation. In the analyzed TF4 and TF5 brines, the occurrence of anaerobes suggested that anoxia episodes may likely occur or had occurred within brines, especially TF5, representing an important regulator of microbial energetics. From the obtained results, another two interesting aspects emerged: the influence exerted by both upward movement of saline brine from a sub-surface anoxic system and the possible occurrence of an ancient ice remnant from the Ross Ice Shelf, both potentially shaping the microbial communities.

Direct measurements, in terms of prokaryotic cell quantification, virus like particles’ abundance and cell viability, provided evidence of the diversification existing between the two brine pockets. Both the physiological profiles and enzymatic potentials displayed diverse metabolic patterns and different contributions to biogeochemical processes between neighboring brines. The detection of discrete levels of β-GLU activity in TF4 compared to TF5 brine suggested the presence, at moderate depths, of organic substrates, providing to the microbial communities, an available C source. Furthermore, the high activity rates of this enzyme and the high numbers of viable cells found in TF4 brine corroborated the presence of an active metabolism, even under these extreme conditions. In addition, the substantially similar LAP and AP activity rates measured in TF brines led us to consider that there were no significant spatial differences in the hydrolytic processes inherent to N and P biogeochemistry driven by the microbial communities. 

This study provides further insights into the ecology and evolution of the microbial life in the ice-covered lakes of Antarctica, coping with the natural extreme brine environment. From an astrobiological perspective, brines could represent a useful and plausible terrestrial model to study the microbial colonization of exobiological niches, encouraging investigation on the existence of life of other planets. 

## Figures and Tables

**Table 1:** Morphometrical and morphological features of the prokaryotic cells in the brines TF4 and TF5.

Feature		TF4	TF5
cell length (µm)	*mean ± sd*	0.543 ± 0.200	0.832 ± 0.580
	*min*	0.32	0.21
	*max*	1.04	3.01
cell width (µm)	*mean ± sd*	0.342 ± 0.08	0.432 ± 0.140
	*min*	0.21	0.21
	*max*	0.53	0.95
mean cell VOL (µm^3^)	*mean ± sd*	0.040 ± 0.027	0.105 ± 0.104
total biovolume (µm^3^ mL^−1^)	*mean ± sd*	0.20 ± 0.11	0.85 ± 0.42
coccobacilli (µm^3^)	*mean ± sd*	0.058 ± 0.021	0.101 ± 0.084
cocci (µm^3^)	*mean ± sd*	0.029 ± 0.019	0.083 ± 0.107
rods (µm^3^)	*mean ± sd*	0.040 ± 0.034	0.113 ± 0.117
curved rods (µm^3^)	*mean ± sd*	n.d.	0.213 ± 0.098

**Table 2:** The raw substrate utilization with absorbances over 0.10 OD recorded at plate level, referred to as averaged substrate color development (ASCD) with time, and the number of positive wells (S) (a). The diversity measures of substrate utilization of the brine samples determined at the time of maxima ASCD value (b).

	TF4		TF5	
(a)	ASCD	S	ASCD	S
17/11/2014	0.070	6	0.085	9
19/11/2014	0.073	8	0.022	4
21/11/2014	**0.075**	9	0.035	5
23/11/2014	0.055	8	0.042	4
25/11/2014	0.021	3	0.038	4
27/11/2014	0.016	1	0.038	4
29/11/2014	0.059	10	0.050	7
01/12/2014	0.060	8	0.047	5
03/12/2014	0.068	**11**	0.079	11
05/12/2014	0.069	**11**	0.096	15
07/12/2014	0.058	10	0.216	25
09/12/2014	0.061	**11**	0.341	28
11/12/2014	0.059	8	**0.542**	**31**
(b)	*21/11/2014*		*11/12/2014*	
*Richness_S*	*9*		*31*	
*Shannon_H*	*2.910*		*3.395*	
*Evenness_e^H/S*	*0.592*		*0.962*	
*Equitability_J*	*0.847*		*0.989*	

**Table 3:** Mean values ± standard deviation of the bulk maximum rates of enzymatic hydrolysis V_max_, cell-specific enzymatic activities and reciprocal molar ratios of the enzymatic activities measured in the two brine samples.

		TF4	TF5
V_max_	LAP	2.04 ± 0.72	1.97 ± 0.25
(nmol L^−1^ h^−1^)	AP	1.05 ± 1.26	1.03 ± 0.09
	β-GLU	2.53 ± 3.90	0.12 ± 0.09
Cell-specific	LAP	0.41 ± 0.02 × 10^−8^	2.44 ± 0.05 × 10^−9^
activity	AP	2.10 ± 0.04 × 10^−9^	1.28 ± 0.02 × 10^−9^
(amol cell^−1^ h^−1^)	β-GLU	0.50 ± 0.02 × 10^−8^	1.44 ± 0.03 × 10^−10^
C:P		1.51 ± 0.31	0.21 ± 0.01
C:N		1.09 ± 0.54	0.16 ± 0.01
N:P		1.38 ± 0.04	1.37 ± 0.03

Abbreviations: LAP, leucin aminopeptidase; AP, alkaline phosphatase; β-GLU, β-glucosidase; C:P, ratio obtained from ln(β-GLU):ln(AP); C:N, ratio obtained from ln(β-GLU):ln(LAP); N:P, ratio obtained from ln(LAP):ln(AP).

## Supplementary Materials

Supplementary materials can be found at https://www.mdpi.com/2076-2607/7/9/333/s1.
