# Supplementary material for: Microbial Assemblages in Pressurized Antarctic Brine Pockets (Tarn Flat, Northern Victoria Land): A Hotspot of Biodiversity and Activity
Source: Microorganisms. 2019 Sep 9;7(9):333. doi: 10.3390/microorganisms7090333 (PMC6780602; doi:10.3390/microorganisms7090333)
Supplement: Supplementary file 1 [file microorganisms-07-00333-s001.pdf]

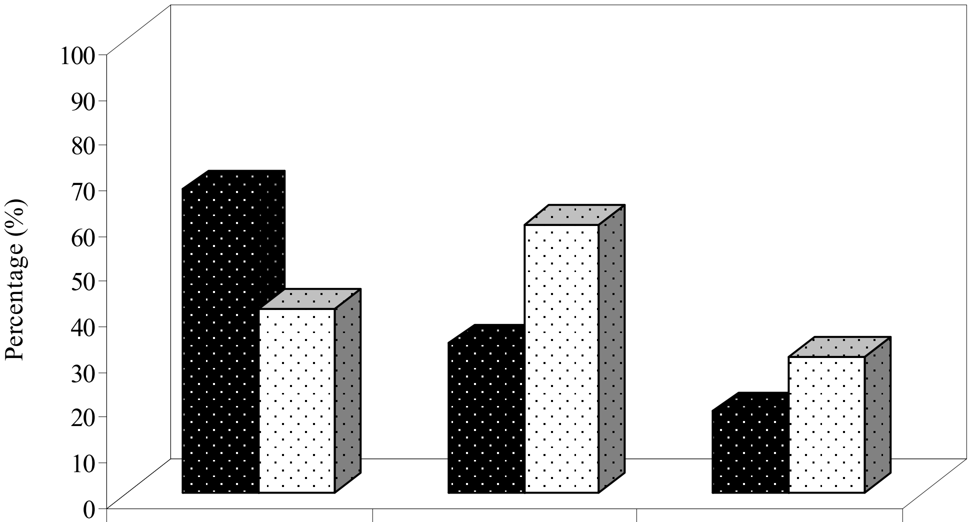

	LIVE	DEAD	CTC
TF 4	67	33	18
TF 5	41	59	30

Supplementary Figure S1. Alive and dead cells (L/D) and respiring cells (CTC+) as percentages of total abundance in TF4 and TF5 bines.

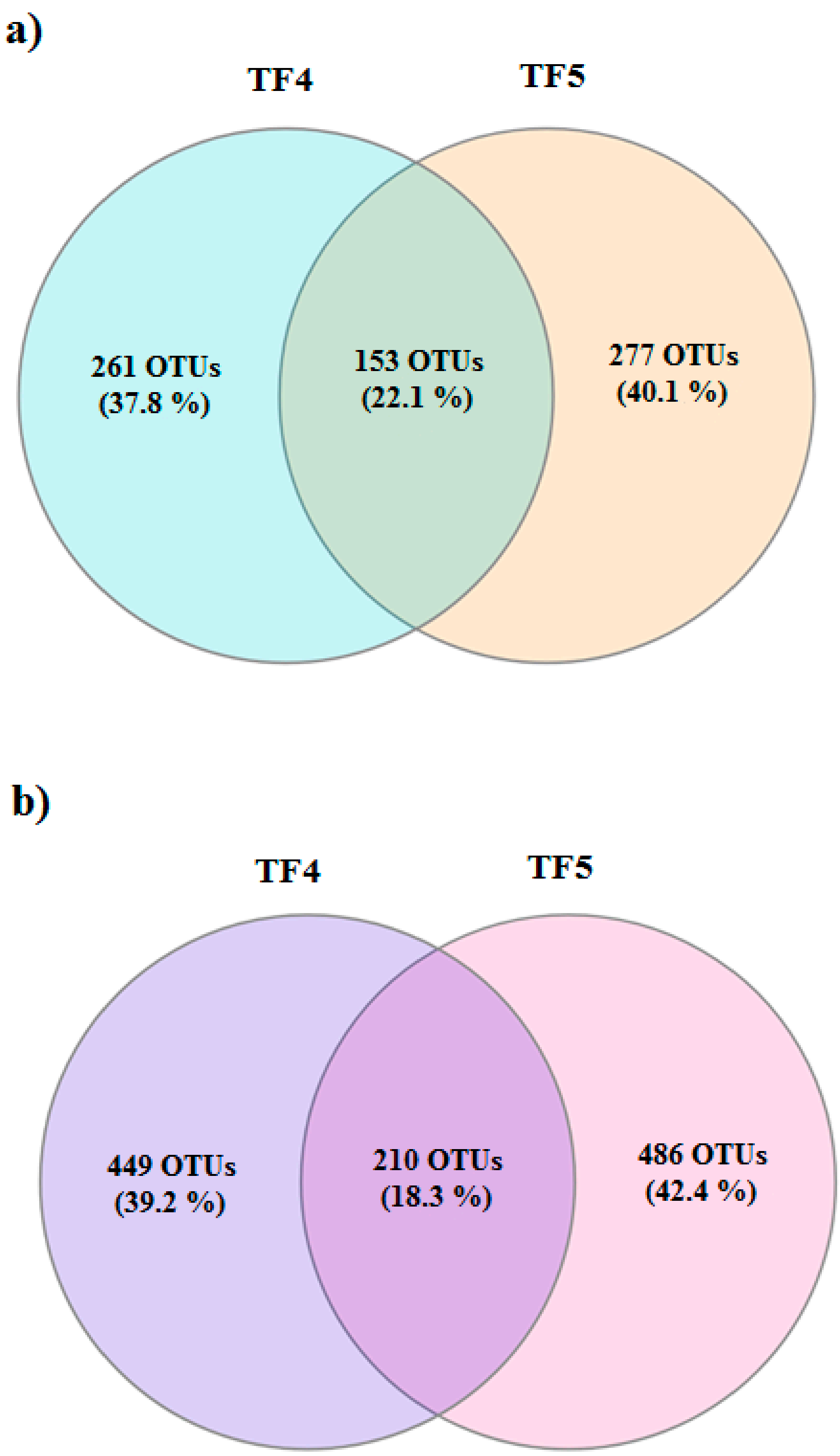

Supplementary Figure S2. Venn diagrams showing the number of bacterial **a)** and archaeal **b)** OTUs shared between brine samples.

Supplementary Table S1. Bacterial genera retrieved in TF4 and TF5 brines.

	TF4	TF5
Phylum or Class	Genus	Genus
Alphaproteobacteria	*Devosia*	*Hoeflea*
	Paracoccus	*Sneathiella*
	Sphingopyxis	*Sphingopyxis*
	Sulfitobacter	*Parvibaculum*
	Roseovarius	*Catellibacterium*
		Pelagibulus
		Devosia
		Paracoccus
		Roseovarius
Betaproteobacteria	*Caenimonas*	*Aquimonas*
	Polaromonas	*Polaromonas*
	Rhodoferax	*Pseudacidovorax*
Gammaproteobacteria	*Haliea*	*Balneatrix*
	Modicisalibacter	*Citrobacter*
	Pseudospirillum	*Haliea*
	Paraferrimonas	*Colwellia*
	Candidatus *Ruthia*	*Shewanella*
	Marinobacter	*Thiomicrospira*
	Thiomicrospira	
	Marichromatium	
Deltaproteobacteria	Candidatus *Thiobios*	*Desulfobulbus*
		Desulfobacula
		Desulfocapsa
		Desulfobacterium
		Desulforhopalus
		Geopsychrobacter
Epsilonproteobacteria	*Sulfurimonas*	*Sulfurimonas*
Actinobacteria	*Yonghaparkia*	*Kytococcus*
	Nitriliruptor	*Microcella*
	Salinibacterium	*Salinibacterium*
	Ornithinicoccus	*Illumatobacter*
	Cariobacterium	*Leifsonia*
	Microcella	Candidatus *Aquilina*
	Lamia	*Okibacterium*
	Okibacterium	
	Physiococcus	
	Illumatobacter	
	Leifsonia	
	Candidatus *Aquiluna*	
Aquificae	*Hydrogenobaculum*	
Bacteroidetes	*Lutimonas*	*Crocinitomix*
	Bizionia	*Flavobacterium*
	Polaribacter	*Psychroflexus*
	Cyclobacterium	*Arenibacter*
	Fluviicola	*Algoriphagus*
	Cellulophaga	*Gaetbulibacter*
	Lutibacter	
	Sediminicola	
	Psychroserpens	
	Dyadobacterium	
	Arenibacter	
	Flavobacterium	
	Gaetbulibacter	
	Psychroflexus	
	Algoriphagus	
	Ulvibacter	
Spirochaetes	*Cloacamonas*	*Spirochete*
Firmicutes	*Alkalibacter*	*Anaerovorax*
	Filifactor	
Planctomycetes	*Schlesneria*	
	Candidatus *Kuenenia*	
Cyanobacteria	*Symploca*	*Geovibrio*

0.1-0.9%
1.0-4.9%
≥5.0%

Supplementary Table S2. Archaeal genera retrieved in TF4 and TF5 brines.

	TF4	TF5
Phylum or Class	Genus	Genus
Crenarchaeota	*Acidilobus*	*Acidilobus*
	Aeropyrum	*Caldisphaera*
	Ignicoccus	*Aeropyrum*
	Acidianus	*Ignicoccus*
	Sulfolobus	*Sulfurisphaera*
		Caldivirga
		Pyrobaculum
		Thermoproteus
		Vulcanisaeta
Euryarchaeota	*Haloarcula*	*Ferroglobus*
	Halobaculum	*Halobacterium*
	Halorhabdus	*Halorhabdus*
	Methanobrevibacter	*Methanobacterium*
	Methanothermus	*Methanothermobacter*
	Methanomicrobium	*Methanothermus*
	Methanoplanus	*Methanoplanus*
	Methanimicrococcus	*Methanosaeta*
	Methanosalsum	*Methanimicrococcus*
	Methermicoccus	*Methanosalsum*
	Thermococcus	*Methanopyrus*
		Thermococcus
		Picrophilus

0.1-0.9%
1.0-4.9%
≥5.0%
